# Host susceptibility to severe COVID-19 and establishment of a host risk score: findings of 487 cases outside Wuhan

**DOI:** 10.1186/s13054-020-2833-7

**Published:** 2020-03-18

**Authors:** Yu Shi, Xia Yu, Hong Zhao, Hao Wang, Ruihong Zhao, Jifang Sheng

**Affiliations:** State Key Laboratory for Diagnosis and Treatment of Infectious Diseases, National Clinical Research Center for Infectious Diseases, Collaborative Innovation Center for Diagnosis and Treatment of Infectious Diseases, The First Affiliated Hospital, College of Medicine, Zhejiang University, Qingchun Road, No. 79, Hangzhou, 310003 China

**Keywords:** COVID-2019, Disease severity, Risk factors, Host susceptibility

The recent outbreak of coronavirus disease 2019 (COVID-19), caused by a new zoonotic coronary virus, SARS-CoV-2 [1], is being a great threat to public health. Up to February 11, 2020, it is reported that over 70,000 persons have been infected with SARS-CoV-2 in China [2]. The COVID-19 caused by SARS-CoV-2 infection represents a spectrum of clinical severity [3–5]. Some patients are asymptomatic or have merely mild upper respiratory tract symptoms. However, SARS-CoV-2 causes pneumonia that can be severe and characterized by fever, cough, dyspnea, bilateral pulmonary infiltrates, and acute respiratory injury. It is estimated that approximately 20% of patients are developing severe respiratory illness, with the overall mortality around 2.3% [2]. Thereby, it is critical to identify individuals who confer intrinsic susceptibility to become severe or even critically ill upon infection, for the purposes of prevention and treatment, especially when there is no drug directly targeting at SARS-CoV-2 that has been proven to be clinically effective. In the study, we explored potential host risk factors associated with severe cases at admission in a retrospective cohort of 487 patients in Zhejiang Province of China and attempt to establish a score system to identify high-risk individuals. We reviewed medical records, laboratory findings, and pulmonary CT scan of each patient with COVID-19, provided by the local health authority and inputted into a pre-specified electronic data collection form. Clinical outcomes were followed up to February 17, 2020. The primary endpoint was occurrence of death and severe cases.

A total of 487 COVID-19 patients were included for analysis, with 49 (10.1%) severe cases at admission. As shown in Table 1, severe cases are elderly (56 (17) vs. 45 (19), *P* < 0.001), with more male (73.5% vs. 50.9%, *P* = 0.003). They have a higher incidence of hypertension (53.1% vs. 16.7%, *P* < 0.001), diabetes (14.3% vs. 5.0%, *P* = 0.009), cardiovascular diseases (8.2% vs. 1.6%, *P* = 0.003), and malignancy (4.1% vs. 0.7%, *P* = 0.025), and less exposure to epidemic area (49.0% vs. 65.1%, *P* = 0.027), but more infected family members (*P* = 0.031). On multivariate analysis, elder age (OR 1.06 [95% CI 1.03–1.08], *P* < 0.001), male (OR 3.68 [95% CI 1.75–7.75], *P* = 0.001), and presence of hypertension (OR 2.71 [95% CI 1.32–5.59], *P* = 0.007) are independently associated with severe disease at admission, irrespective of adjustment of time to admission.

**Table 1:** Demographic, epidermiological characteristics, and underlying comorbidities of patients with confirmed 2019-nCoV infection

Variables	Total (*N* = 487)	Mild (*N* = 438)	Severe (*N* = 49)	*P* value
Age (years)	46 (19)	45 (19)	56 (17)	< 0.001
Sex				
Male	259 (53.2%)	223 (50.9%)	36 (73.5%)	
Female	228 (46.8%)	215 (49.1%)	13 (26.5%)	0.003
Occupation				
Agricultural worker	140 (28.7%)	122 (27.9%)	18 (36.7%)	
Self-employed	219 (45.0%)	203 (46.3%)	16 (32.7%)	
Employee	82 (16.8%)	79 (18.0%)	3 (6.1%)	
Retired	38 (7.8%)	26 (5.9%)	12 (24.5%)	
Student	8 (1.6%)	8 (1.8%)	0 (0%)	< 0.001
Smoking history				
Yes	40 (8.2%)	34 (7.8%)	6 (12.2%)	
No	434 (89.1%)	391 (89.3%)	43 (87.8%)	
Unknown	13 (2.7%)	13 (2.7%)	0 (0%)	0.331
Comorbidities				
Hypertension	99 (20.3%)	73 (16.7%)	26 (53.1%)	< 0.001
Diabetes	29 (6.0%)	22 (5.0%)	7 (14.3%)	0.009
Cardiovascular disease	11 (2.3%)	7 (1.6%)	4 (8.2%)	0.003
Malignancy	5 (1%)	3 (0.7%)	2 (4.1%)	0.025
Chronic liver diseases	22 (4.5%)	20 (4.6%)	2 (4.1%)	0.877
Chronic renal diseases	7 (1.4%)	5 (1.1%)	2 (4.1%)	0.101
Others	32 (6.6%)	27 (6.1%)	5 (10.2%)	0.279
Exposure to confirmed cases	186 (38.2%)	173 (39.5%)	13 (26.5%)	0.077
Family cluster				
0	392 (80.5%)	352 (80.4%)	40 (81.6%)	
1	67 (13.8%)	63 (14.4%)	4 (8.2%)	
2	12 (2.5%)	12 (2.7%)	0 (0%)	
≥ 3	16 (3.3%)	11 (2.5%)	5 (10.2%)	0.031
Recent travel or residence to/in epidemic area	309 (63.4%)	285 (65.1%)	24 (49.0%)	0.027
Time from onset of symptom to admission	2 (3)	2 (3)	3 (5)	0.10

Data are expressed as mean ± standard deviation (SD), median (interquartile range), or number (percent). Comparisons between mild and severe cases were performed by the Mann-Whitney *U* test or a chi-square test

Then, we defined a host risk score on the basis of the three risk factors, to assess the intrinsic host susceptibility to develop severe cases of COVID-19 (Fig. 1a). As shown in Fig. 1b, a step-wise increase in the incidence of severe COVID-19 at admission was observed with the increment of the host risk score (*P* < 0.001). The performance of the score was also validated in 66 patients who presented mild at admission and were under follow-up during hospital stay. Fifteen patients progressed to severe COVID-19 within a median follow-up time of 15 days. No death was reported by the end of follow-up. A similar trend to the above was confirmed when analyzing the correlation between host risk score and occurrence of severe COVID-19 (*P* = 0.014) (see Fig. 1c).

**Fig. 1:**
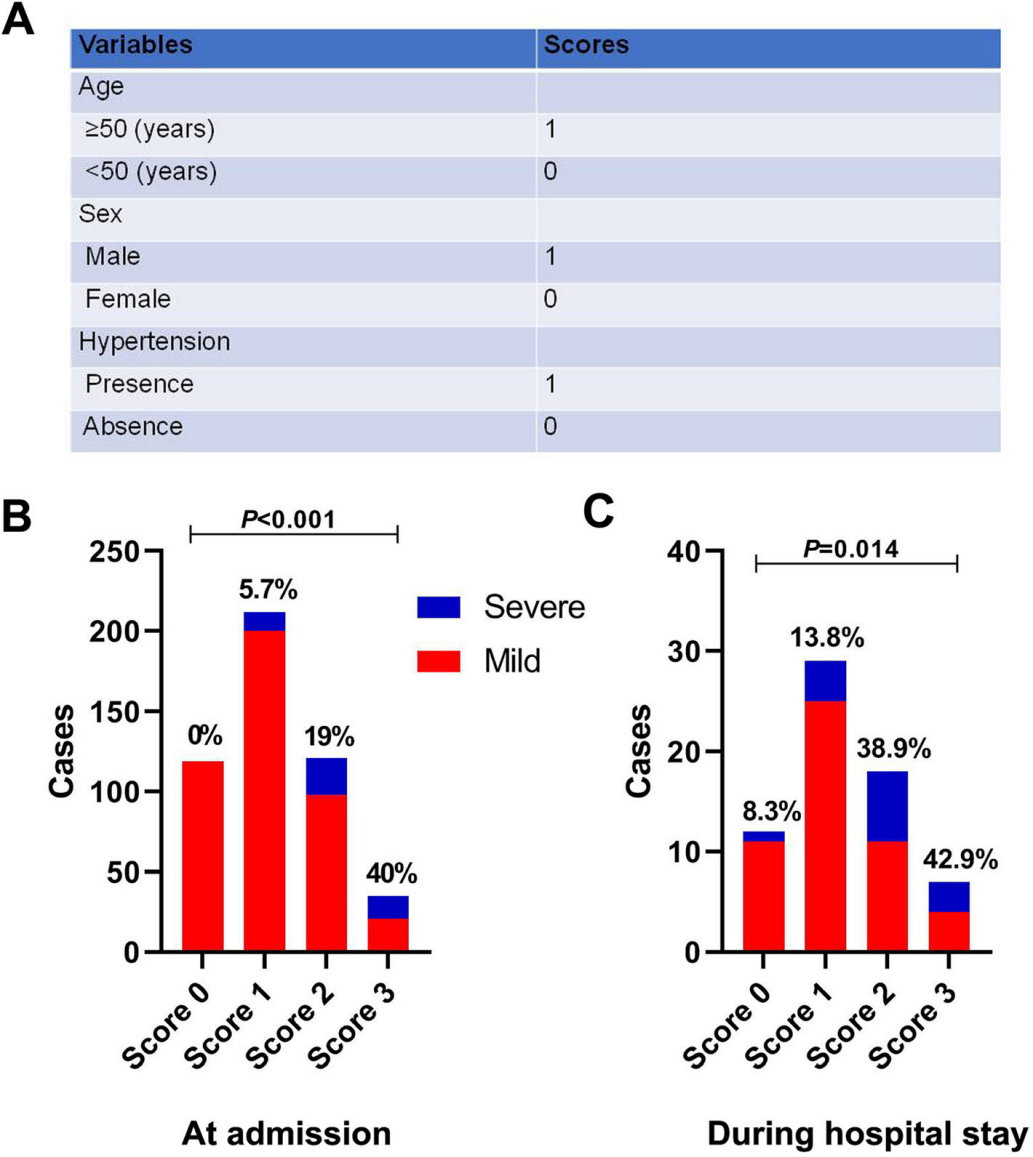
Definition of host risk factor score and incidences of severe cases by host risk score. The host risk factor score was calculated by the sum of three variables (**a**). The incidences of severe cases at admission (**b**) or developing during hospitalization (**c**) were compared across the different score groups by a linear-by-linear association test

In summary, by identifying host risk factors associated with severe COVID-19, this study shed light on the underlying mechanisms of disease progression. In particular, the major finding that hypertension is a host risk factor for severe COVID-19 may underscore the involvement of renin-angiotensin system (RAS) in the pathogenesis of this disease. Additionally, the host risk score provides a useful tool to identify high-risk individuals, which is helpful for designing specific strategies for prevention and treatment of this disease. But further studies, particularly those enrolling Wuhan patients, are needed to validate the findings.

## Data Availability

The datasets and materials used and/or analyzed during the current study are available from the corresponding author on reasonable request.
